# Dietary Melatonin and Glycine Decrease Tumor Growth through Antiangiogenic Activity in Experimental Colorectal Liver Metastasis

**DOI:** 10.3390/nu13062035

**Published:** 2021-06-13

**Authors:** Mindaugas Kvietkauskas, Viktorija Zitkute, Bettina Leber, Kestutis Strupas, Philipp Stiegler, Peter Schemmer

**Affiliations:** 1 General, Visceral and Transplant Surgery, Department of Surgery, Medical University of Graz, Auenbruggerplatz 2, 8036 Graz, Austria; min.kvietkauskas@gmail.com (M.K.); viktorijazitkute@gmail.com (V.Z.); bettina.leber@medunigraz.at (B.L.); peter.schemmer@medunigraz.at (P.S.); 2 Faculty of Medicine, Vilnius University, Ciurlionio 21, 03101 Vilnius, Lithuania; kestutis.strupas@santa.lt

**Keywords:** melatonin, glycine, colorectal cancer, liver metastases, anticancer drug combination

## Abstract

Despite multimodal treatment strategies, clinical outcomes of advanced stage colorectal cancer (CRC) patients remain poor. Neoadjuvant/adjuvant chemotherapy efficacy is limited due to chemoresistance, toxicity, and negative side effects. Since both melatonin and glycine have anti-cancer activities without relevant side effects, this study was designed to investigate their combined effects in experimental CRC liver metastases. CRC metastasis with CC531 cells were induced in male Wistar rats. Melatonin and glycine alone or their combination were supplemented for 14 days (*n* = 100). Blood parameters, a micro-computed tomography scan (tumor volume over time), and immunohistochemistry for Ki67 and CD31 expression in tumor tissue were compared between groups. Melatonin and glycine alone significantly reduced the tumor volume by 63.2% (*p* = 0.002) and 43% (*p* = 0.044) over time, respectively, while tumor volume increased by 8.7% in the controls. Moreover, treatment with melatonin and glycine alone reduced the tumor proliferation index. Most interestingly, the combination therapy did not have any influence on the above-mentioned tumor parameters. The leukocyte count was significantly increased with melatonin at the end of the experiment (*p* = 0.012) which was due to a high lymphocytes count. Tumor microvascular density was significantly reduced in all treatment groups. The results of this study suggest an inhibitory function for melatonin and glycine alone in the case of CRC liver metastasis growth by acting as natural antiangiogenic molecules, followed by angiogenesis-dependent cancer proliferation and immunomodulation.

## 1. Introduction

Colorectal cancer (CRC) is the third most common diagnosed malignancy in men and the second in women, accounting for about 10% of all cancer types worldwide [1]. Patterns and trends in growing CRC incidence correlate with the increasing adoption of Western lifestyles, while the mortality rate declined with improved early detection and prevention through polypectomy [2,3]. Genetic background and a range of modifiable environmental/lifestyle factors, such as being overweight, diet, smoking, physical activity, etc., play a role in CRC etiology [2,4]. Outcomes vary widely based on cancer-specific molecular features, location, and patient characteristics [5].

Despite the significant improvement in therapy over the last decade, patients with metastatic disease have a dismal 5-year survival rate of less than 10% [6]. Liver metastasis is the main site of distant spread and represents the major cause of death in this group of patients [7]. Approximately 25% of patients will have CRC liver metastases (CRLM) at the time of primary diagnosis, while another 25% patients will develop CRLM within 5 years from the first diagnosis [8]. Surgical resection remains the treatment method of choice for isolated CRLM with 5- and 10-year survival rates at around 40% and 25%, respectively [9]. However, only about 20% of patients are suitable for upfront surgery due to non-resectability based on insufficient future liver remnant, which should be around 20–25% of the total functional liver volume [10,11,12]. Other techniques such as radiofrequency or microwave ablation are reserved for patients unfit for surgery [13,14].

The multimodal approach with effective chemotherapy (CTx) has led to more patients benefiting from surgery after reducing the size of CRLM and allowing oncological resection on patients who were previously inoperable [11]. Currently, FOLFOX (infusional 5-fluorouracil, oxaliplatin, and leucovorin), FOLFIRI (infusional 5-fluorouracil, leucovorin, and irinotecan), or CAPOX (infusional capecitabine and oxaliplatin) combined with various monoclonal antibodies against the vascular endothelial growth factor (VEGF) or epidermal growth factor receptor (EGFR) are standard CTx regimens for patients with CRC and CRLM [1,15,16,17]. However, CRC cells are known to lose susceptibility to CTx drugs, and CTx is limited due to toxic side effects [7]. Therefore, further evaluation of novel drug combinations and additives is necessary to improve patient survival.

Melatonin (N-acetyl-5-methoxytryptamine) is an indolic hormone secreted primarily by the pineal gland of human and mammals in response to darkness [18]. Recent studies revealed anticancer actions of melatonin in almost every stage of tumor occurrence and progression through the join action of multiple mechanisms, which renders it a promising therapeutic option for various types of cancer, including CRC [7,19,20].

Glycine, the simplest natural amino acid, is involved in various metabolic and pathophysiological processes important in the growth and survival of proliferating cells, including cancer cells [21,22,23]. Thus, the glycine-related pathways represent an attractive target for the development of anticancer therapeutics. Previously our research group demonstrated that dietary glycine inhibits the growth of CRLM via antiangiogenic properties, while CTx effectiveness remained stable [24,25].

The aim of this study was to assess potential anticancer effects, such as antiangiogenic and antiproliferative, of both melatonin and glycine for CRLM treatment in a rat model.

## 2. Materials and Methods

### 2.1. Animals

A total of 100 male Wistar rats (six-week-old; weight 200–250 g) were used in this study. The animals were obtained from Janvier Labs (Le Genest-Saint-Isle, France) and arrived at the research facility at least 6 days prior to the first intervention. Rats were housed four animals per cage in a controlled environment (22 ± 1 °C; 12 h/12 h light/dark cycle) and had access to fresh water and chow ad libitum. The study followed the guidelines for the handling and care of experimental animals issued by the Federation of European Laboratory Animal Science Associations (FELASA), was approved by the Austrian Federal Ministry of Science, Research and Economy (BMWF-66.010/0141-V/3b/2018), and was conducted according to the 3Rs.

### 2.2. Animal Groups and Experimental Design

**Figure 1:**
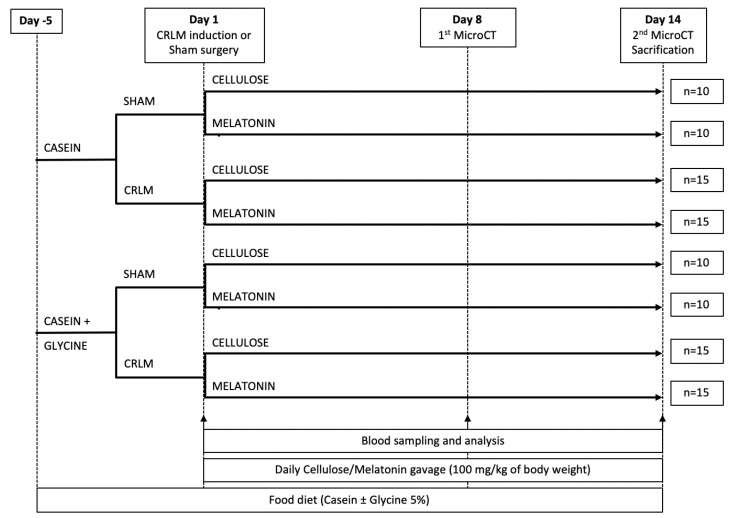
Experimental design. CRLM, colorectal cancer liver metastasis; CT, computed tomography.

The rats were randomly assigned to either sham groups (*n* = 10/group) or experimental groups (CRLM; *n* = 15/group). After a 7-day acclimatization period, the diet for half of the animals in each group was changed to 5% glycine-enriched diet (containing 15% casein and 5% glycine), while the others received a control diet (containing 20% casein and 0% glycine), purchased from Altromin International (Lage, Germany). After 6 days of the special diet, CRLM was induced (experimental groups) or a sham surgery was performed (sham groups), as described below. Rats received 1.5 mL of milk (3.5% fat) containing either melatonin (100 mg/kg of rat body weight; Sigma-Aldrich, St. Louis, MO, USA) or the corresponding amount of microcrystalline cellulose (placebo; from Sigma-Aldrich, St. Louis, MO, USA) daily via gavage. Seven days after CRLM induction, first an abdominal micro-computed tomography (microCT) scan was performed, as described below. After an observation period of another 5 days, a second abdominal microCT scan was performed followed by sacrification and terminal blood and tissue samples collection for further investigation. Water consumption, food intake, and body weight were recorded regularly. The detailed scheme of the experimental model is presented in Figure 1.

### 2.3. CC-531 Cell Line Maintenance

The rat colon adenocarcinoma cell line CC-531 (Cell Lines Service, Eppelheim, Germany) was cultured at 37 °C under a 5% CO_2_ atmosphere. The growth media comprised RPMI 1640 growth medium (GE Healthcare Life Sciences, Logan, UT, USA), supplemented with 10% heat inactivated fetal bovine serum, 2% L-glutamine, 1% penicillin–streptomycin, and 25 mM HEPES buffer. The medium was renewed every other day, and cells were passaged once they grew to approximately 95% confluence.

### 2.4. Tumor Implantation and Sham Surgery

**Figure 2:**
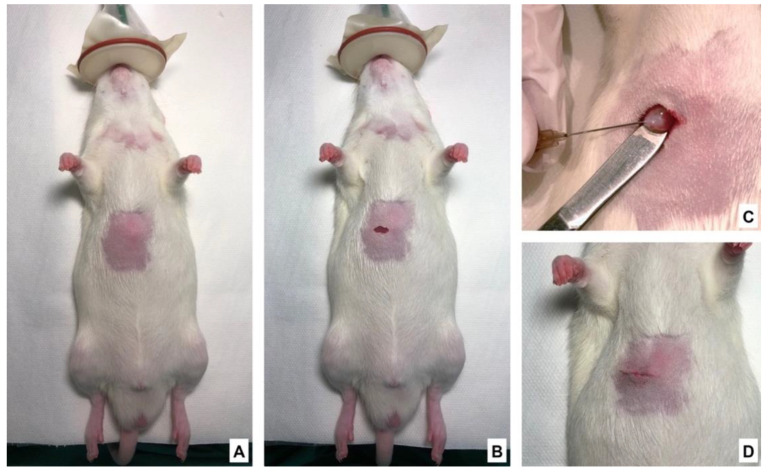
Induction of colorectal cancer liver metastasis: surgical procedure. (**A**) Tumor implantation was performed under general anesthesia; (**B**) Small subcostal incision on the right side of the abdomen; (**C**) Injection of tumor cells under the liver capsule (whitish protrusion in the injection site was the sign for successful inoculation); (**D**) Closed incision.

Anesthesia was performed by 2%, 2 L/min isoflurane inhalation and intramuscular injection of fentanyl (5 µg/kg). Animals were placed in a supine position on an automatically regulated heating pad to maintain normothermia during intervention (Figure 2A). After shaving the right subcostal area, a horizontal laparotomy (measuring about 1 cm) approximately 0.5 cm below the edge of the right ribs was made (Figure 2B). The median liver lobe was exposed, and 100 µL of cell suspension (5 × 10^7^ CC531 cells/mL of phosphate-buffered saline (PBS)) were injected directly under the hepatic capsule using a 26 G needle. Successful inoculation was verified visually (Figure 2C). Compression with a sterile swab to stop bleeding and prevent potential backflow/leakage was applied. Once hemostasis was achieved, a two-layer closure (muscular and subcutaneous) of the wound was performed (Figure 2D) followed by a layer tissue adhesive for skin closure. Immediately after the operation, all animals received 4 mg/kg carprofen and 0.02 mg/kg buprenorphine by subcutaneous injection. For the following 5 days, drinking water was supplemented with 2.5 mg/100 mL tramadol. After medication withdrawal, rats were regularly observed to make sure there were no signs of chronic pain. Sham surgery (control groups) was performed in the same manner as described above but instead of cell suspension, 100 µL sterile PBS were used.

### 2.5. MicroCT Scanning Procedure and Analysis

All rats received a tail vein injection (under 2% isoflurane anesthesia) of the contrast medium ExiTron Nano 12,000 (Viscover, nanoPET Pharma GmbH, Berlin, Germany) 24 h prior to the first microCT scanning procedure. The particular timing of contrast agent administration was selected on the basis of our previous studies [23,24]. An abdominal microCT scan was performed on day 8 (one week after cancer cells’ implantation) and repeated at the end of the experiment on day 14. The single injection of the contrast medium was sufficient to ensure good tumor visibility for the entire duration of the study. Images were obtained using Siemens Inveon microCT scanner (Siemens Medical Solutions, Ann Arbor, MI, USA) with a 0.5 mm filter at 70 kV X-ray voltage, 500 μA anode current, and 800 ms exposure time. After induction of anesthesia with 5% isoflurane, the rat was placed in a prone position on the rat bed. During the scanning period (approximately 23 min), anesthesia was maintained by a further 1.5%–2% isoflurane inhalation via mask. Data were reconstructed using a modified Feldkamp filtered back-projection in Siemens Inveon Acquisition Workplace version 2.1.272 (Siemens Medical Solutions, Ann Arbor, MI, USA). The reconstructed datasets were exported to DICOM format using Siemens Inveon Research Workplace 4.2 software (Siemens Medical Solutions, Ann Arbor, MI, USA) for further analysis.

The microCT image data were analyzed using the commercially available visualization software Mimics Research version 21.0.0.406 (Materialise NV, Leuven, Belgium) by a single examiner. The size and volume of the contrast-enhanced liver and tumor were measured by creating a mask with defined upper and lower threshold ranges (-650 HU and -247 HU for the liver and -806 HU and -609 HU for the tumor, respectively). The mask was then split into separate masks by manually marking each of the regions (liver, non-liver, and tumor) in at least 12 representative views in the transverse plane. Furthermore, a 3D model was created using the “Calculate Part” function. Change in tumor volume was expressed as

$$\mathrm{tumor}\;\mathrm{volume}\;\mathrm{on}\;\mathrm{day}\frac{14\;\left({\mathrm{mm}}^{3}\right)}{8\;\left({\mathrm{mm}}^{3}\right)}\times 100\%$$

### 2.6. Blood Sample Analysis

At experimental day 1 and 8, blood samples were collected from the subclavian vein under 2% isoflurane anesthesia. After the last abdominal microCT scan (day 14), rats were euthanized by intramuscular injection of xylazine (20 mg/kg) and ketamine (100 mg/kg), immediately followed by terminal blood collection from the inferior vena cava. A V-Sight Analyzer (A. Menarini Pharma GmbH, Vienna, Austria) was used to determine a complete blood count. Blood cells were separated from serum/plasma at 1970 xg at 4 °C for 10 min and subsequently stored at −80 °C. Determination of serum glycine levels at the end of the experiment was performed in the routine hospital laboratory.

### 2.7. Immunohistochemical Staining

**Figure 3:**
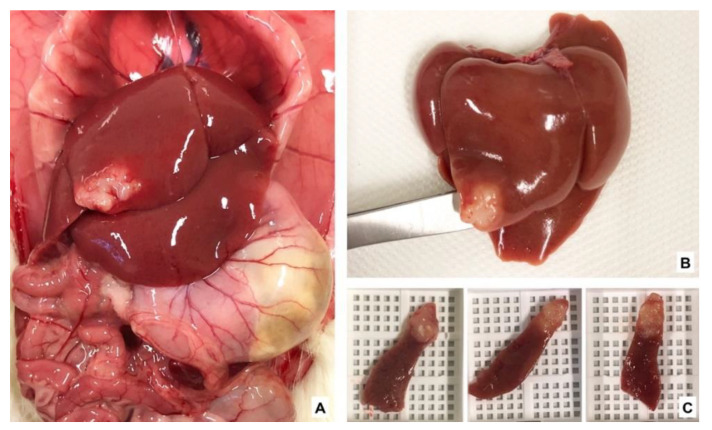
Colorectal cancer liver metastasis and samples for immunohistochemistry. (**A**) Abdominal cavity showing the liver and the implanted tumor; (**B**) Tumor in the median liver lobe; (**C**) Tumor samples for histology.

For immunohistochemistry, tissue samples including the tumor and healthy liver were collected on the last experimental day (Figure 3) and prepared according to the standard procedures. Tissue sections (3 μm thick) were stained with anti-Ki67 antibodies (Thermo Fisher Scientific, Waltham, MA, USA; dilution 1:200, rabbit IgG Clone SP6) to evaluate the tumor proliferation index and with anti-CD31 antibodies (Abcam, Cambridge, UK; dilution 1:2000) to evaluate microvascular density (MVD) in the tumor. For both staining procedures, the UltraVision LP Detection System HRP Polymer (Thermo Fisher Scientific, Waltham, MA, USA) was used in combination with DAB Chromogen (Dako, Via Real Carpinteria, CA, USA) and counterstained with Hematoxylin. For positive controls, rat intestinal and heart tissues were used for Ki67 and CD31, respectively, while for negative controls, primary antibodies were omitted. Stained slides were scanned and analyzed using the QuPath software version 0.2.0-m5 (Belfast, Northern Ireland) [26,27]. For Ki67 analysis, the number of positive cells was counted in a blinded fashion and expressed as the percentage of stained cells of total nuclei. Single endothelial cells or a cluster of cells positive for CD31 were considered as a vessel. MVD was expressed as

$$\frac{\mathrm{number}\;\mathrm{of}\;\mathrm{vessels}\;}{\mathrm{analyzed}\;\mathrm{tumor}\;\mathrm{area}\;\left({\mathrm{mm}}^{2}\right)}\times 10^{-4}$$

### 2.8. Statistical Analyses

Statistical analyses were performed using SPSS version 23.0 (IBM Corp., Armonk, NY, USA). Kruskal–Wallis or Mann–Whitney U tests were used to analyze statistical differences between groups according to their distribution. The data are presented as median and quartiles (Q1; Q3). A *p* value less than 0.05 was considered statistically significant.

## 3. Results

### 3.1. General Data

**Figure 4:**
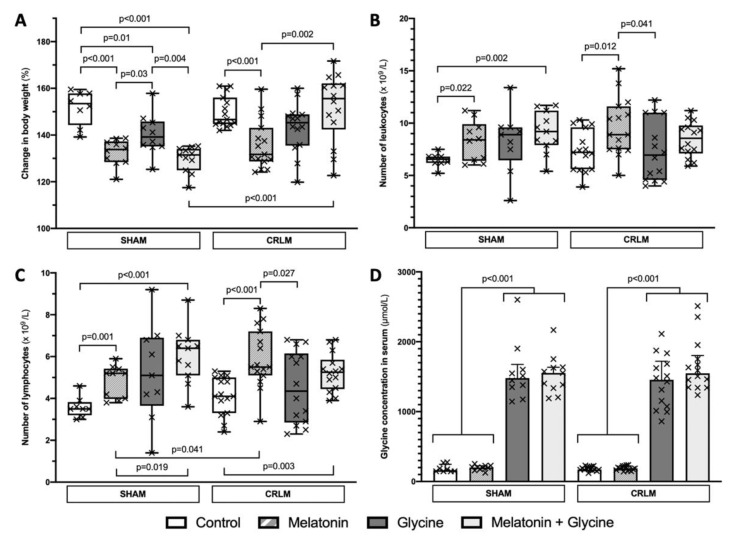
Change in body weight, number of leukocytes and lymphocytes, and glycine concentration in serum at the end of experiment. (**A**) Change in body weight was expressed as ratio of body weight (g) at the end and beginning of the study × 100% in study groups; (**B**) Number of leukocytes at the end of the study in study groups; (**C**) Number of lymphocytes at the end of the study in study groups; (**D**) Glycine concentration in serum at the end of the study in study groups. Data presented as median and interquartile range. Each cross mark represents individual case. CRLM; colorectal cancer liver metastasis.

Ninety-eight (98%) rats underwent the procedure as planned; two animals dropped out prematurely because of problems with anesthesia (*n* = 1) and the postoperative period (*n* = 1). The median body weight of rats increased from 231 (216; 255.5) g at the beginning of the study to 338 (315.5; 352.5) g at the end. Although the average daily food intake was similar, melatonin supplementation significantly reduced the body weight gain compared to the control (133.8 (128.4; 137.3) vs. 153.5% (144.3; 157.8), *p* < 0.001) and glycine (139.2% (135.1; 145.8), *p* = 0.03) in sham groups, and compared to the control (131.7 (128.7; 143.1) vs. 146.6% (144.7; 156.1), *p* < 0.001) and melatonin + glycine (155.6% (142.4; 162.1), *p* = 0.002) in the CRLM groups (Figure 4A). Moreover, glycine supplementation resulted in a reduction in body weight gain in the sham cohort (*p* = 0.01), but not in CRLM (145.3% (135.5; 148.9), *p* = 0.055) when compared to controls. The presence of the tumor itself did not affect body weight gain, except for the CRLM combined treatment group, where it was increased (131.5 (124.9; 134.1) vs. 155.6% (142.4; 162.1), *p* < 0.001).

### 3.2. Blood Test Results and Glycine Concentration

In sham groups, supplementation with melatonin alone and in combination with glycine was associated with an increased number of leukocytes at the end of experiment (*p* = 0.022 and *p* = 0.002, respectively) (Figure 4B and Table 1). CRLM rats treated with melatonin alone had a significantly higher number of leukocytes compared to the control (*p* = 0.012) and glycine (*p* = 0.041) groups, while supplementation with melatonin and glycine did not reach significance (*p* = 0.111). This increase in leukocytes was associated with an increased number of lymphocytes in all groups (Figure 4C and Table 1). The presence of the tumor itself did not impact leukocyte and lymphocyte counts, except CRLM rats supplemented with melatonin had significantly higher numbers of lymphocytes compared to the corresponding sham group (*p* = 0.041), while the combined supplementation of melatonin and glycine did not reach significance (*p* = 0.082).

A glycine-enriched diet increased the median serum glycine concentration by 8.1-fold (186.6 (160; 212.8) vs. 1506.6 (1339.2; 1676.2) µmol/L, *p* < 0.001) as compared to the control, casein diet (Figure 4D and Table 1). The presence of the tumor itself and the combined supplementation with melatonin and glycine did not have an effect on glycine levels.

### 3.3. Change in Tumor Volume

**Figure 5:**
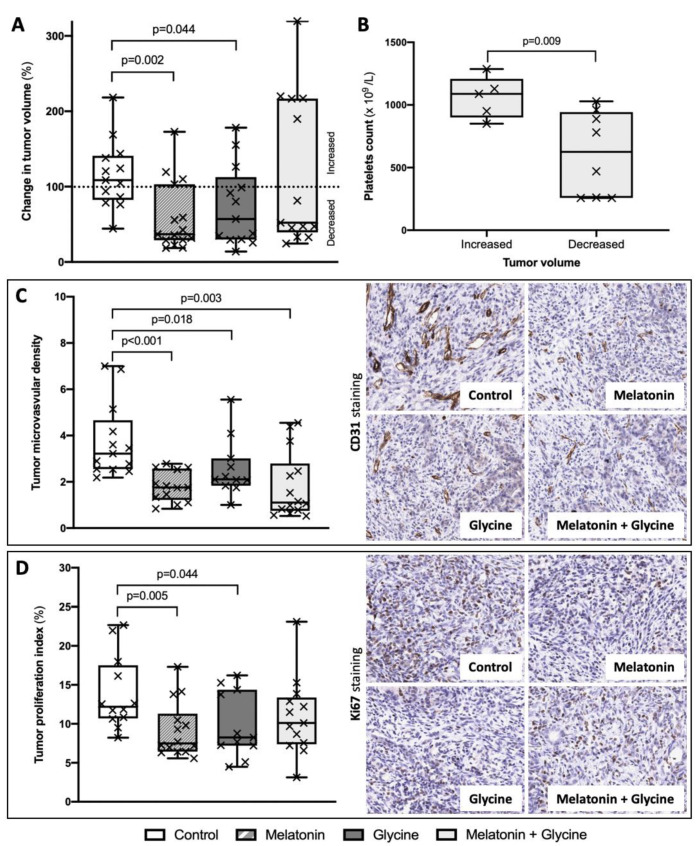
Tumor characteristics and subgroup analysis. (**A**) Change in tumor volume was expressed as tumor volume on day 14 (mm^3^)/tumor volume on day 8 (mm^3^) × 100%; (**B**) Number of platelets before tumor implantation in combined treatment with melatonin and glycine group subgroups according tumor volume change during study period (increase/decrease); (**C**) Tumor microvascular density (MVD) was expressed as number of vessels and tumor area (mm^2^) ratio × 10^−4^ based on immunohistochemistry images of CD31; (**D**) Tumor proliferation index in study groups based on immunohistochemistry images of Ki67. Data presented as median and interquartile range. Each cross mark represents individual case.

We observed an 8.7% (−17.5; 40.9) increased tumor volume in the control group and a 63.2% (−3.1; 71.1) decreased tumor volume in melatonin, 43% (−12.6; 70.1) in glycine, and 47.7% (−116.9; 60.6) in combined supplementation with melatonin and glycine at day 8 vs. day 14. Melatonin and glycine supplementation alone significantly reduced the tumor volume compared to the control group (*p* = 0.002 and *p* = 0.044, respectively), while combined supplementation was found to be non-significant (Figure 5A and Table 2). Moreover, tumor volume was similar in groups supplemented with melatonin, glycine, and their combination.

### 3.4. Tumor Microvascular Density (MVD) and Proliferation Index

Rats supplemented with melatonin, glycine, and their combination had significantly lower MVD in tumor tissue compared to the control group (*p* < 0.001, *p* = 0.018, and *p* = 0.003, respectively) (Figure 5C and Table 2). There was no difference between the groups with respect to the supplementation substance.

The lowest tumor proliferation index has been found in melatonin and glycine groups. Both treatments were associated with a reduced tumor proliferation index compared to controls (*p* = 0.005 and *p* = 0.044, respectively), while the combined treatment with melatonin and glycine did not reduce it significantly (Figure 5D and Table 2). There was no difference when comparing the different supplementation regimens.

### 3.5. Subgroup Analysis: Combined Treatment with Melatonin and Glycine

Subgroup analyses were performed between those animals with increased (*n* = 5) and decreased tumor volumes (*n* = 8) in this group (Figure 5A). The median change in tumor volume was 216.8 (47.6; 219.9) vs. 46.4% (32.9; 51.1) (*p* < 0.001). Tumor volume at day 8 (92.5 (58.5; 213.3) vs. 55.4 (30.2; 216.5) mm^3^, *p* = 0.699) and glycine concentration in serum (1506.6 (1344.5; 1793) vs. 1527.4 (1369.8; 2183.4) µmol/L, *p* = 0.539) were similar between these two subgroups. We found a significantly increased number of platelets before tumor implantation (1088 (900; 1208) vs. 625.5 (258; 942.8) × 10^9^/L, *p* = 0.019) in the cases of tumor enlargement (Figure 5B). Moreover, the subgroup with tumor enlargement had a higher tumor MVD (3.8 (2.4; 4.5) vs. 0.8 (0.6; 1.1) number of vessels/area (mm^2^) × 10^−4^, *p* < 0.001) and proliferation index (14.6 (13.1; 21.2) vs. 8.7% (6.9; 10.8), *p* < 0.001).

## 4. Discussion

Herein, the effects of melatonin alone and in combination with glycine were investigated in the model of CRLM. The results clearly show that supplementation with melatonin or glycine alone significantly reduces the growth of CRLM.

For several years, scientists have been looking for strategies to improve tumor response to treatment and reduce toxicity of drugs. The available data suggest anticancer effects of melatonin and glycine, both in vivo and in vitro, on various different cancers, including CRC [7,24]. However, the combination of these two natural and nontoxic molecules has not been investigated as a potential anticancer modality. Moreover, the effect of melatonin has not yet been analyzed in the model of CRLM, which represents the major cause of death in this type of disease. In this study, we assessed potential anticancer effects of melatonin and glycine for CRLM treatment in an experimental rat model.

We observed that treatment with high-dose melatonin resulted in a slight reduction in body weight gain over the study period. This corresponds to previously published data reporting melatonin to be able to regulate metabolic activity and adipogenesis in mammals and humans [28,29,30]. Hypotheses have been implicated in the explanation of this effect, including indirect effects of melatonin in altering gonadal steroids, adipokines, and glucocorticoid release, as well as the activation of the sympathetic nervous system acting directly on fat depots, regulating the amount of fat stored or mobilized from the adipose tissue [30].

In our model, melatonin significantly reduced tumor growth by 63% in a short time. This could be explained by the immunomodulatory and antiangiogenic properties of melatonin. Supplementation with melatonin was associated with increased numbers of leukocytes, particularly lymphocytes. There is evidence suggesting that lymphocytes play a central role in the host’s response to a tumor [31]. Cytotoxic lymphocytes, cytotoxic T cells, and natural killer cells are the main players in this process, since exocytosis of their granules (perforin and granzymes) and death ligands initiate the most potent pathways used to kill cancer cells, overcoming antiapoptotic mutations, including p53 deletion/mutation, overexpression or downregulation of members of the Bcl-2 family, and caspase inhibition [32].

We found that melatonin reduced tumor vascularization. According to the literature, melatonin employs a variety of mechanisms to target nutrients and oxygen supply to cancer cells. At the transcriptional level, hypoxia induced factor-1α (HIF-1α) and the genes under its control, such as VEGF, which functions as the most important angiogenesis growth factor that promotes cancer progression, are the main targets of melatonin for inhibition of angiogenesis [33,34,35]. Additionally, melatonin reduces endothelin-1, which acts as a survival factor in CRC, expression and secretion in CRC cells through the inactivation of FoxO-1 and NF-κβ, leading to angiogenesis inhibition, thereby limiting development and progression of CRC [36,37].

We were able to confirm the results of our previous experiment, in which dietary glycine decreases tumor volume and vascularization in a combined CRLM and chemotherapy model [24]. In this study, we used a genetically similar rat strain for the CRLM model. We found that treatment with glycine alone significantly decreased tumor volume by 43% over time. This decrease was associated with a reduced MVD and proliferation index in tumor tissue. It is known that glycine blunts VEGF-stimulated endothelial cells’ growth, migration, and angiogenesis via mechanisms involving receptor-dependent pathways [22,24,25].

To validate further our results that dietary melatonin and glycine decrease tumor growth through antiangiogenic activity, additional analyses of VEGF and endothelin-1 expression in tumor tissue should be carried out in the future. Moreover, deeper knowledge about pharmacokinetics of both these substances is needed to evaluate clinically important application regimens.

The tumor volume declined in the majority of rats in the combined supplementation group; however, in some rats an unexpected significant increase in tumor volume, tumor MVD, and proliferation index was observed. We exclude performance bias related to the tumor implantation procedure, including cell batch, as a possible explanation for this unexpected observation. Moreover, tumor size at the first microCT and serum glycine levels at the end of experiment were similar in all animals of this group. Thus, it might be related to the TNF-α effects of both substances. Glycine has been shown to decrease TNF-α, and thus it reduces proliferation of the cells [38,39,40]. In contrast, melatonin is known to attenuate TNF-α expression [41] which obviously counteracts the anticancer properties of glycine in small tumors used in our experimental design.

We found that the number of platelets prior to tumor implantation was significantly higher in the animals whose tumor volume increased. A recent meta-analysis revealed that thrombocytosis is associated with the development and progression of CRC [42]. There is evidence that platelets might be involved in the process of tumor angiogenesis and that numbers of platelets significantly correlate with serum VEGF levels in cancer patients [43]. We believe that, especially in this model, with angiogenesis playing an important role immediately after implantation of tumor cells, increased numbers of platelets release significantly more proangiogenic factors, therefore promoting tumor progression.

If tumor volume reaches a critical level, vascularization is required to omit necrosis and to sustain further tumor growth. This critical volume may not have been reached in our experiments, and therefore it can be speculated that angioneogenesis with the combination therapy was decreased, as in the therapy with single substances without impact on tumor volume. Taking this into account, after single substance therapy, angioneogenesis most likely does not have an impact on tumor volume since tumors were even smaller as compared to the combination therapy, which raises the question about the essential underlying anticancer mechanisms of glycine and melatonin.

Furthermore, biological variability and drug interaction could not be excluded. Until now, data concerning the combination of melatonin and glycine as supplementation in a cancer setting are scarce, making further research mandatory.

## 5. Conclusions

This study suggests an inhibitory function for melatonin and glycine alone in the case of CRLM growth by acting as natural antiangiogenic molecules, with a following angiogenesis-dependent cancer proliferation and immunomodulation. In spite of the fact that dietary melatonin and glycine given separately exert anticancer effects, the combined supplementation with these nontoxic substances did not yield additive effects. To explain these findings, further investigations are warranted.

## Figures and Tables

**Table 1:** Detailed data about leukocytes and lymphocytes count and glycine concentration in serum at the end of the study in study groups.

	SHAM				CRLM			
	Control	Melatonin	Glycine	Melatonin + Glycine	Control	Melatonin	Glycine	Melatonin + Glycine
Leukocytes (× 10^9^/L)	6.5 (6.2; 6.8)	8.4 (6.4; 9.9)	8.9 (6.5; 9.6)	9.2 (7.9; 11.2)	7.2 (5.6; 9.6)	8.9 (7.5; 11.6)	7 (4.6; 11)	8.6 (7.1; 9.8)
Lymphocytes (× 10^9^/L)	3.5 (3.2; 3.8)	5.2 (4; 5.4)	5.1 (3.7; 6.9)	6.4 (5.1; 6.8)	4.1 (3.3; 5)	5.5 (5.1; 7.2)	3.4 (2.9; 6.2)	5.3 (4.5; 5.9)
Glycine (µmol/L)	161.1 (146; 247.2)	201 (171.8; 225.1)	1480.4 (1307.1; 1675.6)	1553.9 (1336.8; 1634.9)	171.1 (167; 210.7)	188.8 (162.6; 221.7)	1455.5 (1058.2; 1721.1)	1548.3 (1345.6; 1803.5)

Data presented as median and quartiles (Q1; Q3). CRLM; colorectal liver metastasis.

**Table 2:** Comparison of tumor characteristics in different treatment groups.

	Control	Melatonin	Glycine	Melatonin + Glycine
Change in tumor volume (%)	108.7 (82.5; 140.9)	36.8 (28.9; 103.1)	56.9 (29.9; 112.6)	52.3 (39.4; 216.9)
Tumor MVD (number of vessels/area (mm^2^) × 10^−4^)	3.2 (2.5; 4.7)	1.8 (1.2; 2.6)	2.1 (1.8; 3)	1.1 (0.7; 2.8)
Tumor proliferation index (%)	12.2 (10.7; 17.5)	7.5 (6.4; 11.3)	8.3 (7.2; 14.4)	10.1 (7.4; 13.4)

Change in tumor volume was expressed as tumor volume on day 14 (mm^3^)/tumor volume on day 8 (mm^3^) × 100%. Data presented as median and quartiles (Q1; Q3). MVD; microvascular density.

## Data Availability

The data that support the findings of this study are available from the first author upon reasonable request.
